# Prevalence of Internet Addiction Among Thai Pharmacy Students: A Cross-Sectional Study

**DOI:** 10.3390/ijerph23040499

**Published:** 2026-04-14

**Authors:** Titawadee Pradubkham, Thuksaorn Sukket, Suphakorn Pimcharee, Kittisak Wichaiyo, Wiraphol Phimarn

**Affiliations:** 1 Clinical Pharmacy Research Unit, Faculty of Pharmacy, Mahasarakham University, Mahasarakham 44150, Thailand; titawadee.p@msu.ac.th; 2 Social Pharmacy Research Unit, Faculty of Pharmacy, Mahasarakham University, Mahasarakham 44150, Thailand; 64010710004@msu.ac.th (T.S.); 64010710071@msu.ac.th (S.P.); 3 General Medicine Department, Kalasin Hospital, Kalasin 46000, Thailand; kittisak.wi.63@ubu.ac.th

**Keywords:** internet addiction, pharmacy students, depression, mental health, cross-sectional study

## Abstract

**Highlights:**

**Public health relevance—How does this work relate to a public health issue?**

- Internet addiction represents an emerging behavioral health issue among university students, driven by increasing reliance on digital technologies in academic and daily life.
- Pharmacy students constitute a high-risk group due to intensive academic demands and prolonged screen exposure, making this population highly relevant for public health investigation.

**Public health significance—Why is this work of significance to public health?**

- A substantial proportion of students exhibited problematic internet use, with 75.95% at risk and 17.47% meeting criteria for internet addiction, indicating a considerable public health burden.
- Significant associations between internet addiction, psychological distress, and depressive symptoms underscore the interconnected nature of digital behavior and mental health outcomes.

**Public health implications—What are the key implications or messages for practitioners, policy makers and/or researchers in public health?**

- Universities should monitor students at risk of Internet addiction, particularly those with an increased risk of depressive symptoms, in order to enable early identification of problems and facilitate timely referral to appropriate specialists.
- Policy makers and public health practitioners should develop targeted prevention strategies and strengthen institutional mental health support systems to mitigate the impact of excessive internet use.

**Abstract:**

The internet is increasingly embedded in daily life; however, excessive use may lead to internet addiction, adversely affecting health and academic performance. This study aimed to determine the prevalence of internet addiction and its association with depressive symptoms among pharmacy students at Mahasarakham University. A cross-sectional survey was conducted between 1 October and 31 December 2025 among undergraduate pharmacy students (years 1–6) aged ≥18 years who provided voluntary consent. Data were collected via street-based and online self-administered questionnaires validated for content and reliability. Descriptive and inferential statistics were applied, and binary logistic regression was used to identify associated factors. Among 396 participants, the mean internet use was 9 h 43 min per day. The prevalence of excessive use (risk) was 75.95%, while 17.47% met criteria for internet addiction. High to very high psychological problems were significantly associated with internet addiction (aOR = 3.89; 95% CI: 1.70–8.89; *p* = 0.001). Risk of depression (2Q) was also significantly associated (aOR = 2.67; 95% CI: 1.39–5.15; *p* = 0.003). Internet addiction is strongly associated with mental health factors, highlighting the need for targeted prevention and institutional mental health interventions.

## 1. Introduction

In the contemporary digital landscape, information technology has become an integral component of everyday life, influencing communication, education, professional practice, and leisure activities. Internet utilization is now widespread and deeply embedded across diverse age groups, reflecting its essential role in modern society [1,2]. At present, the Internet functions as a central medium for educational engagement, providing extensive access to global knowledge repositories such as scholarly publications, electronic books, multimedia learning materials, and online courses [3]. This digital environment promotes flexible and autonomous learning, allowing individuals to pursue knowledge at their own pace, independent of temporal and geographical constraints. Beyond its educational applications, the Internet continues to be extensively used for communication and entertainment purposes, further underscoring its pervasive influence in daily life [4].

As of 2025, an estimated 5.56 billion individuals approximately 67.9% of the global population were using the Internet, with around 5.24 billion people (63.9%) actively engaged on social media platforms [5]. Over the past decade, Internet adoption has expanded rapidly, with substantial growth particularly evident among younger populations [6]. The Internet has evolved into an indispensable resource for information retrieval and interpersonal communication, fundamentally shaping how individuals interact and access knowledge.

Despite its numerous benefits, excessive Internet use has emerged as a growing concern, especially among adolescents and young adults [7,8]. Internet addiction is generally characterized by persistent and uncontrolled online engagement that consumes excessive amounts of time, often resulting in a loss of temporal awareness and disruption of daily functioning. This pattern of behavior may contribute to a range of adverse outcomes, including psychological distress, social difficulties, and impaired personal well-being. Increasingly, problematic Internet use is being recognized as a global public health issue [9,10].

Conceptually, Internet addiction refers to a maladaptive pattern of excessive and poorly regulated Internet use that leads to significant impairment in everyday life [11]. Diagnostic frameworks, such as those proposed by Young et al., highlight key features including tolerance, diminished control, preoccupation with online activities, and prolonged duration of use [12]. The prevalence of this condition varies across demographic factors such as age, sex, and ethnicity, but it appears to be particularly prominent among university students.

Furthermore, individuals with problematic Internet use frequently exhibit coexisting psychological and behavioral concerns. These include mood disturbances, impaired sleep quality, impulsivity, low self-esteem, and, in severe cases, suicidal ideation. Physical health consequences such as reduced physical activity, migraine, back pain, and obesity have also been associated with excessive Internet engagement [9,13,14].

Internet addiction has been conceptualized as a form of impulse-control disorder, characterized by persistent and compulsive patterns of online behavior [15]. This condition can be described in terms of four principal features. First, individuals exhibit excessive Internet use, often accompanied by a diminished awareness of time and neglect of routine responsibilities. Second, withdrawal symptoms may arise when Internet access is restricted, manifesting as irritability, psychological distress, or low mood. Third, tolerance develops, whereby increasing amounts of time spent online are required to achieve the same level of satisfaction, often alongside a growing demand for more advanced technological resources. Finally, functional impairment becomes evident, including interpersonal conflicts, deceptive behaviors, declining academic or occupational performance, social withdrawal, and persistent fatigue [16].

Notably, individuals with problematic Internet use are often aware of the negative consequences associated with their behavior; however, they continue to engage excessively, driven by the reinforcing and rewarding aspects of online activities. Although Internet addiction is not formally recognized as a distinct mental disorder in the Diagnostic and Statistical Manual of Mental Disorders, Fifth Edition (DSM-5), it has attracted considerable attention due to emerging evidence of underlying neurobiological alterations and cognitive impairments. These include deficits in executive functioning, such as impaired working memory, which parallel those observed in substance-related and behavioral addictions [9,17,18].

According to the World Health Organization (WHO), mental and behavioral disorders are among the leading contributors to disability worldwide [19]. These conditions encompass a broad spectrum of psychological disturbances, including depression, which is typically characterized by persistent feelings of guilt, worthlessness, irritability, restlessness, and a diminished interest in previously enjoyable activities. University students represent a particularly vulnerable population, as they are navigating a critical developmental transition from adolescence to adulthood. This period is often accompanied by substantial academic, social, and personal pressures, which may predispose individuals to increased levels of stress, anxiety, and depressive symptoms [20,21].

Excessive engagement with the Internet has been increasingly linked to a range of negative outcomes, affecting academic performance, occupational functioning, interpersonal relationships, and overall mental well-being. Evidence from prior research indicates that individuals exhibiting problematic Internet use frequently experience academic difficulties, reduced social interaction, and diminished self-regulation in controlling their online behavior [22]. Furthermore, Internet addiction has been associated with multiple psychiatric manifestations. For example, a study involving university students reported strong associations between Internet addiction and several mental health conditions, including depression (adjusted (a)OR = 5.79; 95% CI: 2.40–13.8), anxiety (adjusted OR = 5.47; 95% CI: 2.39–12.5), aggression (aOR = 4.02; 95% CI: 1.75–9.25), phobic anxiety (aOR = 11.2; 95% CI: 1.57–80.5), and psychotic symptoms (aOR = 3.06; 95% CI: 1.41–6.64) [23].

Emerging evidence suggests that problematic Internet use is notably prevalent among pharmacy students. For instance, a study conducted in Iraq examining the relationship between social media use and depressive symptoms reported that approximately 38% of pharmacy students were at risk of social media addiction [24]. Similarly, research among undergraduate pharmacy students in Egypt identified a prevalence of Internet addiction symptoms of 38.5%. This study also demonstrated significant associations between excessive Internet use and adverse psychological outcomes, including depression, anxiety, and stress, emphasizing the need for targeted preventive and supportive strategies within this population [25].

Further insights are provided by Younes et al. who investigated Internet addiction among students in health-related disciplines, including medicine, dentistry, and pharmacy. In this cross-sectional study involving 600 participants, validated assessment tools were employed, such as the Young Internet Addiction Test (YIAT), Insomnia Severity Index (ISI), Depression Anxiety Stress Scales (DASS-21), and Rosenberg Self-Esteem Scale (RSES). Analytical approaches included descriptive statistics and logistic regression modeling. The results indicated that 16.8% of students exhibited problematic Internet use, with a higher prevalence observed among male participants. Importantly, Internet addiction was significantly associated with multiple domains of psychological distress, including insomnia, depression, anxiety, stress, and reduced self-esteem (*p* < 0.001), highlighting the substantial mental health burden linked to excessive Internet use among university students [26].

Excessive engagement with digital technologies has increasingly been linked to problematic patterns of Internet use, which may negatively influence physical health, psychological well-being, and social functioning. This issue is particularly relevant among university students, who depend heavily on Internet-based resources for academic activities. Pharmacy students, in particular, are required to process complex scientific and clinical information, necessitating frequent and sustained online engagement for literature searching, coursework preparation, and independent learning. Beyond academic demands, the Internet is also widely used for communication and recreational purposes, which may further heighten the risk of developing maladaptive usage behaviors.

Although Internet addiction has been extensively explored in general student populations, studies specifically focusing on pharmacy students remain comparatively scarce. This gap is especially pronounced within the Thai context, where pharmacy education is characterized by distinct curricular structures and academic intensity that may not be fully comparable to those in other settings. As a result, context-specific research is warranted to provide a clearer understanding of the extent and consequences of Internet addiction among pharmacy students in Thailand, while considering the unique academic pressures and learning environments they encounter.

Accordingly, the present study aimed to assess the prevalence of Internet addiction among pharmacy students at Mahasarakham University, to identify factors associated with and contributing to this condition, and to examine its relationship with depressive symptoms within this population.

## 2. Materials and Methods

### 2.1. Study Design and Participants

This study employed a cross-sectional survey design and was conducted among pharmacy students at Mahasarakham University, Thailand, between 1 October 2025 and 31 December 2025. The target population consisted of undergraduate students enrolled in Years 1 through 6 in the Faculty of Pharmacy during the 2025 academic year (B.E. 2568). Inclusion criteria: Participants were considered eligible if they fulfilled the following conditions: (1) current enrollment as a Year 1–6 pharmacy student at Mahasarakham University; (2) age of 18 years or older; (3) access to an electronic device capable of completing an online questionnaire (such as a smartphone, tablet, or computer); and (4) provision of informed consent prior to participation. Exclusion criteria: Responses were excluded from the analysis if the submitted questionnaires were incomplete.

The sample size was determined using a finite population approach, given that the total number of eligible students was known. In the 2025 academic year, the population of pharmacy students (Years 1–6) comprised 616 individuals. The minimum required sample size was calculated using the Taro Yamane formula [27], based on a 95% confidence level and a margin of error of 5% (*e* = 0.05).

The formula is presented as follows:

$$n=\frac{N}{1+Ne^{2}}$$

where *n* represents the required sample size, *N* denotes the total population size, and *e* refers to the acceptable margin of error (set at 0.05). By substituting the corresponding values into the formula (*N* = 616; *e* = 0.05), the minimum sample size was calculated as follows: *n* = 616/[1 + 616(0.05)^2^]. Based on this calculation, the minimum required sample size was determined to be 243 participants. Accordingly, from a total population of 616 students, a sample of at least 243 individuals was considered sufficient to achieve an acceptable level of statistical precision.

### 2.2. Questionnaire and Data Collection

#### 2.2.1. Section 1: Demographic Characteristics and Internet Use Behaviors

This section collected information on participants’ demographic profiles and patterns of Internet use. It consisted of five items addressing demographic variables, including sex, age, religion, year of study, and grade point average (GPA), along with five additional items related to Internet use behaviors and knowledge of Internet-related regulations. These items assessed average daily Internet usage on both weekdays and weekends, primary locations of Internet access, participation in offline recreational activities, typical periods of Internet use during the week, and awareness of legal regulations governing Internet use. Participants’ knowledge regarding Internet-related laws and restrictions was evaluated using a four-point Likert scale ranging from 1 (“no knowledge at all”) to 4 (“high level of knowledge”). Mean scores were interpreted according to predefined categories: 1.00–1.75 indicated no knowledge, 1.76–2.50 reflected low knowledge, 2.51–3.25 represented moderate knowledge, and 3.26–4.00 indicated a high level of knowledge [28].

#### 2.2.2. Section 2: Psychological Problems Assessment

This section comprised eight items designed to evaluate psychological concerns associated with Internet use. The items covered multiple domains, including low self-confidence, dissatisfaction with oneself, negative emotional states, feelings of loneliness or sadness, boredom, emotional instability or heightened emotional intensity, sensation-seeking tendencies, and anxiety. Responses were recorded using a five-point Likert scale, with scores ranging from 1 (“strongly disagree”) to 5 (“strongly agree”). The interpretation of mean scores was categorized into five levels of psychological impact: scores between 1.00–1.80 indicated very low levels, 1.81–2.60 low levels, 2.61–3.40 moderate levels, 3.41–4.20 high levels, and 4.21–5.00 very high levels of psychological problems [28].

#### 2.2.3. Section 3: Social Relationship Problems

This section included six items aimed at evaluating social difficulties associated with Internet use, particularly its impact on interpersonal interactions and overall social functioning. Responses were collected using a five-point Likert scale ranging from 1 (“strongly disagree”) to 5 (“strongly agree”). Mean scores were categorized to reflect the severity of social relationship problems as follows: 1.00–1.80 indicated very low levels, 1.81–2.60 low levels, 2.61–3.40 moderate levels, 3.41–4.20 high levels, and 4.21–5.00 very high levels of social relationship impairment [28].

#### 2.2.4. Section 4: Interpersonal Relationships

This section assessed the quality of interpersonal relationships across two domains: family relationships (9 items) and peer relationships (10 items). Responses were measured using a four-point Likert scale, with scoring defined as 4 = true, 3 = somewhat true, 2 = somewhat untrue, and 1 = not true. Mean scores were interpreted to indicate the level of relationship quality, where scores of 1.00–1.75 represented a low level, 1.76–2.50 a moderate level, 2.51–3.25 a high level, and 3.26–4.00 a very high level of relationship quality with family members or peers [28].

#### 2.2.5. Section 5: Inappropriate Internet Use Behaviors

This section comprised nine items designed to evaluate patterns of inappropriate Internet use. Participants were asked to indicate the frequency of engagement in these behaviors using a four-point Likert scale ranging from 1 to 4. The response options were defined as follows: 4 = very often or regularly (81–100%), 3 = frequently (51–80%), 2 = occasionally (21–50%), and 1 = rarely or never (0–20%). Mean scores were interpreted to reflect the level of engagement in such behaviors, where scores of 1.00–1.75 indicated a low level, 1.76–2.50 a moderate level, 2.51–3.25 a high level, and 3.26–4.00 a very high level of inappropriate Internet use [28].

#### 2.2.6. Section 6: Purpose of Internet Use and Internet Addiction Behaviors

This section assessed both the purposes of Internet use and indicators of Internet addiction, and was divided into two subsections.

Subsection 6.1 consisted of six items exploring the purposes of Internet use through a combination of open-ended and closed-ended questions. The open-ended items captured the estimated duration of Internet use across different activities, while the closed-ended items required respondents to report time ranges and indicate whether the actual time spent was less than intended, as intended, or exceeded their original intention.

Subsection 6.2 included eight items designed to measure Internet addiction-related behaviors across multiple types of online activities. Responses were recorded using a six-point scale ranging from 0 to 5, with scoring defined as follows: 5 = regularly, 4 = very often, 3 = often, 2 = occasionally, 1 = rarely, and 0 = never.

The overall level of Internet addiction behavior was determined by integrating two components: (1) discrepancies between intended and actual time spent online, and (2) the frequency of engagement in Internet-related activities. For the time discrepancy component, spending more time than intended was assigned 3 points for weekdays and 2 points for weekends, whereas spending less time than intended was assigned 0 points for both periods. The behavioral frequency component was scored on a scale of 0 to 5 based on reported usage frequency. The total composite score ranged from 0 to 45 points and was categorized into three levels: scores of 0–22 indicated normal Internet use, 23–35 suggested a risk of Internet addiction, and 36–45 reflected Internet addiction behavior [28].

#### 2.2.7. Section 7: Internet Addiction Assessment (YIAT)

This section employed YIAT, a widely used standardized instrument comprising 20 items to evaluate the severity of problematic Internet use. Responses were recorded on a five-point Likert scale ranging from 1 to 5, resulting in a total score between 20 and 100. Based on established criteria, total scores were classified into three levels: 20–39 indicated normal Internet use, 40–69 suggested excessive use with a potential risk of developing Internet addiction, and 70–100 reflected significant impairment in controlling Internet use, consistent with Internet addiction [29].

#### 2.2.8. Section 8: Depression Assessment (2Q and 9Q)

Depressive symptoms were assessed using the 2-item Depression Screening Questionnaire (2Q) and the 9-item Depression Assessment Questionnaire (9Q), both developed by the Department of Mental Health, Thailand. Initial screening was conducted using the 2Q, which includes two items. Participants who responded positively to at least one item were subsequently evaluated using the 9Q to determine the severity of depressive symptoms. The 9Q instrument employed in this study is a standardized depression screening tool developed by the Thai Department of Mental Health. It represents a culturally adapted version of the original 9-item Patient Health Questionnaire (PHQ-9), which is widely recognized and utilized in international settings. The 9Q preserves the core diagnostic criteria of the PHQ-9 while incorporating linguistic and contextual modifications to ensure appropriateness within the Thai clinical and sociocultural context. The instrument comprises nine items assessing key depressive symptoms over a specified period, including anhedonia; depressed mood; sleep disturbances (e.g., insomnia or hypersomnia); fatigue; appetite changes; negative self-perception (e.g., worthlessness or failure); impaired concentration; psychomotor changes (slowing or agitation); and recurrent thoughts of self-harm or death [30,31]. The 9Q scores were categorized into four levels: scores below 7 indicated no depressive symptoms, 7–12 represented mild depression, 13–18 indicated moderate depression, and scores of 19 or higher reflected severe depression [32].

#### 2.2.9. Instrument Reliability

Reliability was evaluated using Cronbach’s alpha (α) coefficients in a pilot sample of 15 pharmacy students who were not included in the main study. The results demonstrated good internal consistency across all domains, as follows: Computer and Internet Use Behaviors and Legal Awareness (α = 0.82), Psychological Problems Assessment (α = 0.81), Social Relationship Problems (α = 0.81), Family Relationships (α = 0.80), Peer Relationships (α = 0.82), Inappropriate Internet Use Behaviors (α = 0.82), Purpose of Internet Use and Internet Addiction Behaviors (α = 0.84), YIAT (α = 0.90), and Depression Assessment using the 2Q and 9Q (α = 0.89 and 0.92, respectively).

### 2.3. Statistical Analysis

Data were analyzed using Stata software version 15.1 (StataCorp, College Station, TX, USA). The analytical process was carried out in three sequential steps. First, descriptive statistics were applied to summarize participant characteristics and study variables, including demographic information, patterns of Internet use, psychological factors, social relationship indicators, levels of Internet addiction, and depressive symptoms. These findings were reported using frequencies, percentages, means, and standard deviations, as appropriate. Second, differences in Internet use duration across various purposes between weekdays (Monday to Friday) and weekends (Saturday and Sunday) were examined using independent *t*-tests. This approach allowed for comparison of mean usage times according to different usage objectives. Third, the relationships between potential explanatory variables and Internet addiction were explored. Initial univariable analyses were conducted to screen for candidate variables. Factors with a *p*-value less than 0.25 were subsequently included in a multivariable model, following the recommended approach by Zhang (2016) [33]. We conducted a multicollinearity assessment among the variables psychological distress, 2Q, and 9Q using a correlation matrix. The interpretation of correlation coefficients (r) was based on the following criteria: |r| < 0.30 was considered low (no concern), 0.30–0.69 moderate (acceptable), 0.70–0.79 indicative of potential concern, ≥0.80 suggestive of high multicollinearity, and ≥0.90 indicative of severe multicollinearity or redundancy [34]. Multivariable binary logistic regression was then performed to identify independent factors associated with Internet addiction. The results were expressed as adjusted odds ratios (aORs) with corresponding 95% confidence intervals (CIs), and statistical significance was defined at a threshold of *p* < 0.05. All data processing and statistical analyses were conducted using Stata.

## 3. Results

### 3.1. Demographic Characteristics

The demographic profile of the participants is summarized as follows. The majority of respondents were female (*n* = 286, 72.22%). In terms of age, most participants were between 20 and 23 years old (*n* = 301, 76.01%), while a smaller proportion were older than 23 years (*n* = 49, 12.37%). With respect to academic standing, the highest proportion of students were in Year 5 (*n* = 80, 20.20%), followed closely by those in Year 4 (*n* = 77, 19.44%). Regarding religious affiliation, nearly all participants identified as Buddhist (*n* = 383, 96.72%). Academic performance, as reflected by GPAX, showed that the majority of students achieved a GPAX between 3.50 and 4.00 (*n* = 203, 62.46%), followed by those with a GPAX of 3.00–3.49 (*n* = 99, 30.46%). In terms of Internet access, most participants reported using the Internet primarily within the university facilities, such as faculty buildings or computer centers (*n* = 242, 61.27%), while others accessed it through student dormitories (*n* = 130, 32.91%). Further details are provided in Table 1.

### 3.2. Internet Use Patterns

**Figure 1:**
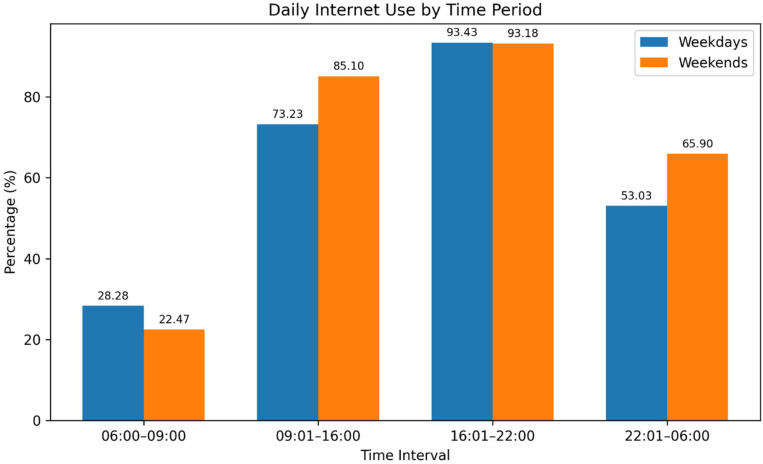
Distribution of Internet use by time intervals, presented as frequency and percentage, during weekdays (Monday–Friday) and weekends (Saturday–Sunday).

The duration of engagement in offline leisure activities was examined according to day type, classified as weekdays (Monday–Friday) and weekends (Saturday–Sunday). On average, participants reported spending 3 h and 31 min per day on non-Internet-related hobbies during weekdays, which increased to 4 h and 52 min per day on weekends. In contrast, the mean daily duration of Internet use was considerably higher, averaging 9 h and 43 min. When analyzed by day type, participants reported spending approximately 8 h and 18 min online during weekdays, rising to 9 h and 42 min per day on weekends. With regard to time-of-day patterns, Internet use was most prevalent during the late afternoon and evening period (16:01–22:00 h) on both weekdays (*n* = 370, 93.43%) and weekends (*n* = 369, 93.18%). The second most common usage period was 09:01–16:00 h, reported by 73.23% of participants on weekdays (*n* = 290) and 85.10% on weekends (*n* = 337) (Figure 1).

**Figure 2:**
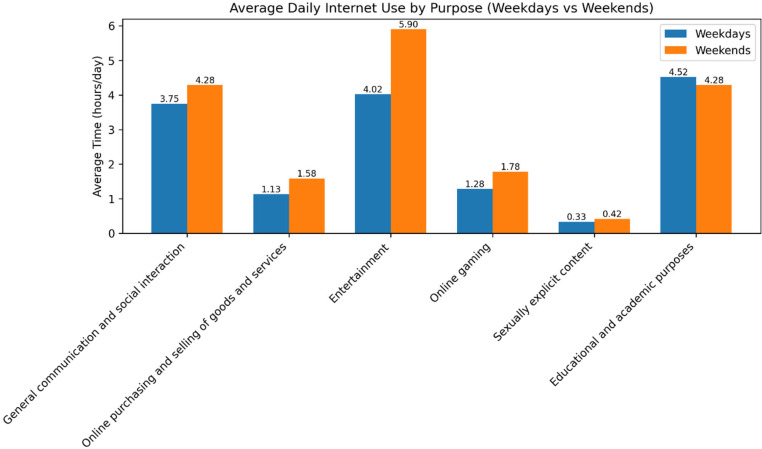
Average duration of Internet use by purpose.

The analysis of Internet use by purpose demonstrated clear differences between weekdays and weekends. During weekdays (Monday–Friday), the longest average duration of Internet use was devoted to educational activities and information seeking (4 h and 31 min), followed by entertainment-related use, such as streaming media, listening to music, or downloading content (4 h and 1 min). Internet use for general communication averaged 3 h and 45 min, while online gaming accounted for a comparatively shorter duration of 1 h and 17 min. In contrast, patterns shifted during weekends (Saturday–Sunday), with entertainment emerging as the most time-intensive activity, averaging 5 h and 54 min. This was followed by both communication and educational purposes, each with an average duration of 4 h and 17 min. Further examination of time-of-day trends revealed consistent patterns across both weekdays and weekends. For communication and social interaction during weekdays, Internet use was most concentrated in the late afternoon and evening period (16:01–22:00 h; *n* = 334, 84.34%), while the lowest level of use occurred in the early morning (06:01–09:00 h; *n* = 79, 19.94%). A similar distribution was observed during weekends, with peak usage for social-related activities also occurring between 16:01 and 22:00 h (Figure 2).

The comparison of mean Internet use duration between weekdays (Monday–Friday) and weekends (Saturday–Sunday), stratified by purpose, revealed notable differences across several categories. Overall, time spent online was significantly lower on weekdays than on weekends for activities such as general communication, online purchasing of goods and services, entertainment (e.g., streaming media, listening to music, or downloading content), online gaming, and accessing sexually explicit material (e.g., pornographic websites or images) (*p* < 0.05).

In contrast, no statistically significant difference was observed in Internet use for educational purposes and knowledge acquisition between weekdays and weekends (*p* > 0.05), indicating relatively consistent usage patterns for academic activities across the week (Table 2).

### 3.3. Computer and Internet Use Behaviors and Legal Awareness

The results indicated that participants exhibited a very high level of appropriate computer and Internet use, with an overall mean score of 3.89. At the item level, the highest mean score was observed for refraining from using a friend’s email password to access private messages (mean = 3.98), followed closely by avoiding unauthorized access to a friend’s computer (mean = 3.97) (Table A1). In contrast, participants demonstrated a relatively low level of awareness regarding legal restrictions and penalties associated with Internet use, with a mean score of 2.18 ± 0.54, suggesting limited knowledge in this domain).

### 3.4. Results of Internet Addiction

Internet addiction among participants was evaluated using Young’s Internet Addiction Test (YIAT) [29], which classifies the severity of problematic Internet use. The overall mean score was 58.13 (SD = 12.35), indicating that, on average, participants fell within the range of excessive Internet use and were considered at risk of developing Internet addiction. Based on the established YIAT classification, the majority of participants were categorized as being at risk of Internet addiction (*n* = 300, 75.95%). In addition, 17.47% of participants met the criteria for Internet addiction, reflecting significant difficulty in controlling their Internet use (Table 3).

### 3.5. The Psychological Problems

The assessment of psychological problems indicated that participants, on average, experienced a moderate level of psychological distress, with a mean score of 2.70. At the item level, higher mean scores were observed in domains related to concern or anxiety about outcomes not meeting expectations (mean = 3.54) and a tendency to seek novel or stimulating experiences (mean = 3.43). In contrast, lower mean scores were identified for dissatisfaction with oneself and current life circumstances (mean = 2.30), irritability and difficulty regulating emotions when expectations were not fulfilled (mean = 2.24), and confidence in expressing opinions or engaging in social interactions (mean = 1.98). Notably, none of the assessed domains reached a very high level of severity (Table A2).

### 3.6. Social Relationship Problems

Overall, participants reported a low level of social relationship difficulties, with a mean score of 2.35. At the item level, only one domain reached a moderate level, namely the desire for more supportive or closer family relationships than currently experienced (mean = 2.71). Several other aspects were rated at a low level, including discomfort when engaging in social interactions (mean = 2.59) and perceived challenges in social adjustment (mean = 2.45). Detailed results for the remaining items are presented in (Table A3).

### 3.7. Family Relationships

Overall, participants reported a very high level of family relationship quality, with a mean score of 3.28. Item-level analysis indicated that several aspects of family relationships were rated at a very high level, including perceptions of love and affection from parents and family members, consistent care and attention, a sense of warmth within the family environment, recognition or praise for positive behaviors, and emotional support during times of personal difficulty. Other dimensions were rated at a high level, such as overall satisfaction with parents and family members, as well as a desire for continued improvement in family relationships. Notably, no participants were classified as having moderate or low levels of family relationship quality. Detailed findings are presented in Table A4.

### 3.8. Peer Relationships

Overall, participants reported a high level of peer relationship quality. At the item level, several aspects were rated at a very high level, including experiencing enjoyment when spending time with friends, receiving encouragement during periods of discouragement, deliberately selecting friends with positive behaviors, and feeling emotionally affected when conflicts or disagreements occurred. Other domains were rated at a high level, such as providing guidance to friends when they were at risk of engaging in inappropriate behaviors, preferring to socialize or travel with friends, and seeking support or advice from friends when encountering personal challenges. Notably, no participants were classified as having low or moderate levels of peer relationship quality (Table A5).

### 3.9. Study Outcomes Related to Depressive Symptoms

The results showed that most participants (72.84%) did not present with depressive symptoms. Mild depressive symptoms were observed in 78 individuals, representing 19.80% of the study population. Detailed distributions of depressive symptom severity are provided in Table 4.

### 3.10. Factors Associated with Internet Addiction

#### Univariable Analysis

The univariable binary logistic regression analysis identified eight factors that were significantly associated with Internet addiction among pharmacy students. This included year of study, categorized academic year, GPAX, psychological problems, social relationship problems, family relationships, and results from the 2-item depression screening questionnaire (2Q) and the 9-item depression assessment questionnaire (9Q). With respect to academic year, a significant association with Internet addiction was observed. Students in Years 2, 5, and 6 demonstrated higher odds of Internet addiction compared with first-year students. Notably, those in Years 5 and 6 were significantly more likely to exhibit Internet addiction than those in Year 1 (*p* = 0.022). When academic years were grouped, students in Years 5–6 had significantly greater odds of Internet addiction compared with those in Years 1–4 (*p* = 0.017). In terms of psychological factors, participants reporting high to very high levels of psychological problems were 4.20 times more likely to experience Internet addiction compared with those reporting very low to moderate levels (*p* < 0.001). Similarly, students with high to very high levels of social relationship problems had significantly increased odds of Internet addiction relative to those with lower levels (*p* = 0.001). Regarding depression screening outcomes, participants identified as being at risk for depression based on the 2Q were more likely to demonstrate Internet addiction than those without such risk. In addition, students with moderate to severe depressive symptoms showed significantly higher odds of Internet addiction compared with those without depression or with only mild symptoms (*p* = 0.016). Detailed results are presented in Table 5.

Following the inclusion of variables that met the screening criterion from the univariable analysis (*p* < 0.25), and after assessing multicollinearity, psychological problems and the 2Q depression screening were selected for inclusion in the multivariable analysis. The results of the correlation matrix showed that psychological distress was weakly correlated with 2Q (r = −0.2651), indicating no concern for multicollinearity, and moderately correlated with 9Q (r = 0.4018), which is considered acceptable. In contrast, a strong correlation was observed between 2Q and 9Q (r = 0.8226; |r| > 0.80), indicating clear multicollinearity and substantial conceptual overlap between these two measures. Due to the high correlation between 2Q and 9Q (r = 0.82), suggesting both conceptual and statistical redundancy, these variables were not included simultaneously in the multivariable model. Based on these findings, psychological distress and 2Q were selected for inclusion in the multivariable analysis. A multivariable binary logistic regression model was subsequently constructed. After adjustment for potential confounders, two factors remained significantly associated with Internet addiction among pharmacy students: psychological problems and the 2Q depression screening results. Students reporting high to very high levels of psychological problems had a 3.89-fold higher likelihood of Internet addiction compared with those reporting very low to moderate levels (adjusted odds ratio [aOR] = 3.89; 95% confidence interval [CI]: 1.40–7.93; *p* = 0.001). In addition, participants identified as being at risk of depression based on the 2Q demonstrated a 3.97-fold increased likelihood of Internet addiction compared with those not at risk (aOR = 3.97; 95% CI: 1.29–4.75; *p* = 0.002). These results are presented in Table 6.

## 4. Discussion

The present study aimed to estimate the prevalence of potential Internet addiction among Thai university pharmacy students, examine its associations with participant characteristics, and explore its relationships with family dynamics, social and peer interactions, and depressive symptoms. The findings indicated that the average duration of Internet use was 8 h and 18 min per day on weekdays, increasing to 9 h and 42 min on weekends. The highest level of Internet activity on both weekdays and weekends occurred during the late afternoon and evening period (16:01–22:00), accounting for 93.43% and 93.18% of usage, respectively. The primary purposes of Internet use were education, entertainment, and social networking.

The results further demonstrated that most participants primarily used the Internet for academic purposes. This pattern may partly reflect the lasting impact of the COVID-19 pandemic, during which many higher education institutions transitioned to online learning modalities to mitigate transmission risks. Although data collection for this study occurred after the acute phase of the pandemic, the structure of the pharmacy curriculum continues to emphasize both individual and collaborative assignments. These academic requirements necessitate frequent use of online platforms for group work, academic discussions, and communication with instructors. Among the platforms reported, Microsoft Teams was the most commonly utilized. As a result, students are likely to spend prolonged periods engaging with digital devices, including tablets and computers, throughout both weekdays and weekends.

Based on classification using YIAT, the mean score in this study was 58.13 ± 12.35. The majority of participants (75.95%) were categorized as exhibiting excessive Internet use or being at risk of Internet addiction, while 17.47% demonstrated impaired control over their Internet use and were therefore classified as having Internet addiction. The mean YIAT score observed in this study was higher than that reported by Sayed (2022) [25], who examined Internet addiction among Egyptian pharmacy students and found a mean score of 44.75, with a prevalence of 38.5%. Consistent with prior research, elevated levels of Internet addiction have also been documented among student populations in various Asian countries [35,36]. For example, studies among adolescents in Taiwan and Germany reported prevalence rates of 49.5% and 71.6%, respectively [37]. The findings of the present study are also comparable to those reported among medical students, where 31.2% were classified as mildly addicted and 12.4% as moderately addicted [38]. Although this study employed the YIAT, which is a widely used and standardized screening instrument, we acknowledge that the YIAT is designed to capture a broad spectrum of problematic Internet use behaviors rather than to establish a formal clinical diagnosis of Internet addiction. Therefore, the prevalence reported in this study likely reflects problematic or maladaptive patterns of Internet use (problematic use spectrum) rather than clinically meaningful addiction.

With regard to associated factors, the univariable analysis indicated that sex was not significantly associated with Internet addiction. This finding differs from earlier studies, such as that by Scherer et al., which identified male sex as a significant predictor. The discrepancy may reflect temporal shifts in Internet use patterns, with gender-related differences becoming less pronounced over time. In contrast, academic seniority appeared to play a more prominent role. Students in the later years of study (Years 5 and 6) were more likely to spend extended periods online compared with those in earlier years (Years 1–4). This may be attributable to the increased academic demands placed on senior students, including more complex coursework, research activities, and collaborative assignments, all of which require frequent and sustained use of Internet-based resources.

Senior pharmacy students, particularly those in their final year, are typically engaged in experiential or clinical training across diverse practice settings, including hospitals, community pharmacies, pharmaceutical industries, and other healthcare environments. These training experiences require the integration of theoretical knowledge with practical clinical application. Importantly, such activities often involve daily case discussions with preceptors, participation in journal clubs, academic presentations, and the provision of drug-related recommendations to healthcare professionals and patients. Consequently, students must frequently access up-to-date clinical information through online databases, clinical guidelines, and digital platforms. Evidence from medical and nursing education supports this explanation, indicating that students in clinical training phases demonstrate higher utilization of digital resources due to the demands of evidence-based decision-making and continuous learning in real-time clinical settings [39,40]. The findings of the present study are consistent with previous research suggesting that intensive academic engagement and prolonged screen exposure may contribute to problematic Internet use. Studies conducted among medical and pharmacy students have shown that frequent Internet use for academic purposes can coexist with maladaptive usage patterns, particularly when students encounter difficulties in maintaining a balance between academic and non-academic activities [38]. Furthermore, excessive Internet use has been associated with a range of adverse outcomes, including poorer academic performance, sleep disturbances, and psychological distress, underscoring the importance of monitoring Internet use within this population [41]. In addition, the present study found that higher levels of psychological distress, social difficulties, and the presence of depressive symptoms were significantly associated with Internet addiction among pharmacy students. These associations remained statistically significant not only in univariable analyses but also after adjustment for potential confounders in multivariable logistic regression models (*p* < 0.05). However, given the cross-sectional design, these findings should be interpreted as correlations observed at a single time point and do not imply causality or directional relationships. Accordingly, psychological factors should be considered as variables associated with problematic Internet use rather than independent factors. These results are consistent with a growing body of international literature demonstrating significant associations between Internet addiction and mental health conditions, including depression, anxiety, and stress. Among these, depressive symptoms have been reported to show a particularly strong association, with higher levels of depression linked to an increased likelihood of problematic Internet use [25,26,42].

This association can be understood within the framework of maladaptive coping theory, which suggests that individuals may engage in excessive Internet use as a strategy to avoid or alleviate negative emotional states. In the context of pharmacy education, students may turn to online activities as a means of coping with academic demands, stress, and depressive symptoms encountered in daily life. Instead of addressing the underlying sources of distress, they may rely on digital entertainment or social media to obtain temporary emotional relief. However, such coping behaviors may lead to unintended negative consequences, including ineffective time management, diminished academic performance, and increased stress accumulation. Over time, these outcomes may further intensify psychological distress, particularly depressive symptoms, thereby reinforcing a cycle in which excessive Internet use becomes a repeated coping response. This reciprocal pattern where emotional distress contributes to increased Internet use, which in turn exacerbates mental health problems has been highlighted in recent studies [42].

A substantial body of evidence has consistently demonstrated a strong association between depression and Internet addiction. For example, Saikia et al. (2019) [43] reported that adolescents with problematic Internet use exhibited significantly higher levels of depression, anxiety, and stress, with depression showing the strongest relationship. Similarly, a meta-analysis by Cai et al. (2023) [44] identified a moderate but consistent correlation between problematic Internet use and depressive symptoms across diverse populations. In higher education settings, studies such as those by Li et al. (2023) [45] have further confirmed that depressive symptoms are significantly associated with Internet addiction among university students. These findings are consistent with the results of the present study, in which depression remained significantly associated with Internet addiction even after adjusting for potential confounding variables.

Beyond depression, a broader range of psychological difficulties including anxiety, stress, and impaired emotional regulation has been identified as contributing to problematic Internet use. A systematic review by Sayed et al. (2022) reported significant associations between Internet addiction and multiple dimensions of psychological distress among students, including depression, anxiety, and stress [25]. In addition, Xie et al. (2023) [46] proposed a mediational framework in which anxiety contributes to Internet addiction, which subsequently intensifies depressive symptoms, suggesting a complex and potentially bidirectional relationship. These findings are consistent with the present study, where individuals reporting higher levels of psychological distress were more likely to exhibit Internet addiction, indicating that such distress may both predispose individuals to and be aggravated by excessive Internet use.

The observed relationship between social difficulties and Internet addiction is also supported by existing literature. Individuals experiencing challenges such as loneliness, interpersonal conflict, or limited social support may engage in excessive Internet use as a compensatory mechanism to fulfill unmet social needs. For instance, Cai et al. (2023) [44] demonstrated that problematic Internet use is significantly associated with loneliness and reduced social well-being. Other studies have similarly suggested that online environments may provide a perceived sense of social connection that may be lacking in offline interactions. These findings align with the current results, where higher levels of social problems were associated with an increased likelihood of Internet addiction.

Importantly, the persistence of these associations in multivariable analyses highlights the independent contribution of psychological factors. For example, Vishwakarma et al. (2021) [47] found that depression and stress remained significant predictors of Internet addiction after adjusting for sociodemographic characteristics. Similarly, Al Saigh et al. (2022) [48] reported that depressive symptoms were independently associated with problematic social media use among university students. Consistent with these findings, the present study demonstrated that psychological problems remained significantly associated with Internet addiction even after controlling for other variables, suggesting that mental health factors represent key determinants rather than merely correlates of problematic Internet use.

### Strengths and Limitations

The findings of this study should be interpreted in consideration of its methodological context and inherent limitations. Data were collected using self-administered questionnaires, a commonly employed approach for assessing subjective aspects of physical and mental health. This method allows participants to report their own perceptions and experiences; however, it may also be subject to reporting bias and variability in individual interpretation. Additionally, the questionnaire included a relatively large number of items, which may have contributed to respondent fatigue. This could have affected participant engagement and resulted in incomplete responses, leading to the exclusion of some questionnaires from the final analysis. Most pharmacy students in this study were high-performing, with over 92% having a GPA above 3.0. Although no significant association was observed between academic performance and Internet addiction, caution should be exercised when generalizing these findings to other populations. Finally, this study was conducted among students from a single faculty within one university, which may limit the external validity and generalizability of the findings. Future research should consider optimizing questionnaire length to improve response rates, data completeness, and overall data quality. Furthermore, the study population was limited to pharmacy students within a single institution, which may restrict the generalizability of the findings. Subsequent studies should aim to include participants from diverse academic disciplines and a broader range of age groups to enhance external validity. Expanding the scope of investigation to include additional factors related to digital device use such as smartphone or tablet dependency and their associated physical and psychological outcomes may also provide a more comprehensive understanding of the issue. Such approaches could contribute to identifying underlying mechanisms and inform the development of more targeted and effective interventions.

## 5. Conclusions

In summary, a substantial proportion of pharmacy students were identified as being at risk of Internet addiction (75.95%), with 17.47% meeting the criteria for Internet addiction based on the Thai version of Young’s Internet Addiction Test (YIAT). The study further demonstrated significant associations between Internet addiction and psychological distress, particularly the risk of depression. These findings highlight the close interplay between problematic Internet use and mental health, emphasizing the need for early detection and continuous monitoring within university populations. A comprehensive approach to prevention and management is warranted, incorporating strategies that address both Internet use behaviors and underlying psychological conditions such as depression, anxiety, and stress. Efforts to raise awareness among students regarding the potential health consequences of excessive Internet use especially its impact on mental well-being are essential. In addition, higher education institutions should consider strengthening mental health support services, including accessible counseling programs and psychiatric care, to better support students at risk. Future research should extend beyond the current scope to examine the relationship between Internet addiction and other risk behaviors, such as substance use and illicit drug involvement, in order to provide a more comprehensive understanding of the multifactorial nature of this condition among university students.

## Figures and Tables

**Table 1:** Demographic Characteristics of the Participants (*n* = 396).

Demographic Data		Number (*n*)	%
Gender			
	Male	110	27.78
	Female	286	72.22
Age (years)			
	<20	46	11.61
	20–23	301	76.01
	>23	49	12.38
	Mean (SD)	22.72 (2.1)	
Academic year			
	1	60	15.15
	2	74	18.69
	3	72	18.18
	4	77	19.44
	5	80	20.20
	6	33	8.33
Religion			
	Buddhism	383	96.72
	Islam	5	1.26
	Christianity	4	1.01
	Other religions	4	1.01
Cumulative Grade Point Average (GPAX)			
	3.50–4.00	203	62.46
	3.00–3.49	99	30.46
	2.00–2.99	22	6.77
	Below 2.00	1	0.31
Primary Location of Internet Use			
	Educational institution (faculty/department/computer center)	242	61.27
	Student dormitory	130	32.91
	Home (living with family)	19	4.81
	Friend’s or another person’s residence	1	0.25
	Internet café/Other locations	3	0.76

**Table 2:** Comparison of Internet use duration by purpose between weekdays (Monday–Friday) and weekends (Saturday–Sunday).

Purpose of Internet Use	Duration of Use (Mean ± SD) (min)		*p*-Value
	Weekdays	Weekends	
For general communication and social interaction	225.74 ± 208.62	257.16 ± 235.39	<0.001
For online purchasing of goods and services	68.16 ± 83.98	96.42 ± 112.82	<0.001
For entertainment purposes (e.g., watching movies, listening to music, or downloading content)	241.38 ± 182.40	354.87 ± 228.40	<0.001
For online gaming	77.42 ± 112.85	108.38 ± 141.05	<0.001
For accessing sexually explicit content (e.g., pornographic websites or images)	20.52 ± 86.54	25.41 ± 90.42	<0.001
For knowledge acquisition and academic study	271.06 ± 202.47	257.97 ± 190.72	0.6866

**Table 3:** Levels of Internet addiction severity.

Severity Level of Internet Addiction	Number (*n*)	%
No Internet addiction	26	6.58%
Excessive Internet use/At risk of Internet addiction	300	75.95%
Internet addiction (inability to control Internet use)	69	17.47%

**Table 4:** Levels of depressive symptom severity.

Severity Level of Depressive Symptoms	Number (*n*)	%
No depressive symptoms	287	72.84
Mild depressive symptoms	78	19.80
Moderate depressive symptoms	25	6.35
Severe depressive symptoms	4	1.02

**Table 5:** Univariable analysis of factors associated with Internet addiction.

Factors		Univariate Analysis		
		OR	95% CI	*p*-Value
Gender				
	Male	1.00	Reference	
	Female	1.25	0.71–2.37	0.37
Academic Year Level				
	First-year students	1	Reference	
	Second-year students	2.74	0.93–8.06	0.066
	Third-year students	1.74	0.55–5.41	0.337
	Fourth-year students	1.80	0.58–5.49	0.302
	Fifth-year students	3.36	1.17–9.62	0.024
	Sixth-year students	4.05	1.22–13.36	0.022
Academic Year Group				
	Years 1–4	1	Reference	
	Years 5–6	1.21	1.02–1.43	0.017
Religion				
	Buddhism	1	Reference	
	Others (e.g., Christianity, Islam, or other religions)	0.94	0.20–4.40	0.941
Cumulative Grade Point Average (GPAX)				
	Less than 3.49	1	Reference	
	3.50–4.00	0.69	0.18–1.20	0.188
Internet Usage Location				
	Outside the educational institution	1	Reference	
	Within the educational institution (faculty/department)	1.28	0.70–2.18	0.465
Psychological Problems				
	Very low to moderate levels	1	Reference	
	High to very high levels	4.20	2.17–8.13	0.001
Social Problems				
	Very low to moderate levels	1	Reference	
	High to very high levels	4.22	1.82–9.77	0.001
Family Relationships Problems				
	Very low to moderate levels	1	Reference	
	High to very high levels	1.40	0.83–2.37	0.197
Friend Relationships Problems				
	Very low to moderate levels	1	Reference	
	High to very high levels	1.26	0.74–2.13	0.379
Internet Use Behavior				
	Low to good levels	1	Reference	
	Very good levels	0.63	0.06–6.16	0.693
Depression Screening (2Q)				
	No risk (no positive response on the 2Q)	1	Reference	
	At risk (≥1 positive response on the 2Q)	2.31	1.36–3.94	0.002
Depression Screening (9Q)				
	No depression to mild depression	1	Reference	
	Moderate to severe depression	2.72	1.20–6.16	0.016

Remark: OR = Odds Ratio; 95% CI = 95% Confidence Interval.

**Table 6:** Multivariable analysis of factors associated with Internet addiction.

Factors		Multivariate Analysis		
		aOR	95% CI	*p*-Value
Academic Year Group				
	Years 1–4	1	Reference	
	Years 5–6	1.62	0.71–3.11	0.470
Cumulative Grade Point Average (GPA)				
	Less than 3.49	1	Reference	
	3.50–4.00	0.84	0.44–1.74	0.695
Psychological Problems				
	Very low to moderate levels	1	Reference	
	High to very high levels	3.89	1.40–7.93	0.001
Social Problems				
	Very low to moderate levels	1	Reference	
	High to very high levels	2.45	0.77–6.10	0.152
Family Relationships Problems				
	Very low to moderate levels	1	Reference	
	High to very high levels	1.19	0.48–2.76	0.792
Depression Screening (2Q)				
	No risk (no positive response on the 2Q)	1	Reference	
	At risk (≥1 positive response on the 2Q)	3.97	1.29–4.75	0.002

Remark: aOR = adjusted Odds Ratio; 95% CI = 95% Confidence Interval.

## Data Availability

The datasets used and/or analyzed during the current study are available from the corresponding author on reasonable request.

## Abbreviations

The following abbreviations are used in this manuscript:

aOR	Adjusted Odds Ratio
CIs	Confidence Intervals
DASS-21	Depression Anxiety Stress Scales
GPA	Grade Point Average
DSM-5	Diagnostic and Statistical Manual of Mental Disorders, Fifth Edition
ISI	Insomnia Severity Index
OR	Odds Ratio
RSES	Rosenberg Self-Esteem Scale
SD	Standard Deviations
WHO	World Health Organization
YIAT	Young Internet Addiction Test
2Q	2-item Depression Screening Questionnaire
9Q	9-item Depression Assessment Questionnaire

## Appendix A

**Table A1:** Descriptive characteristics of computer and Internet use behaviors among participants.

Question	Mean Score	SD	Level
1. Not accessing a friend’s computer without permission.	3.97	0.19	Very high
2. Not secretly viewing data on a friend’s computer without their knowledge.	3.96	0.21	Very high
3. Not downloading music or movies from unauthorized websites that distribute content for free.	3.84	0.40	Very high
4. Not using false names or fraudulent information when registering as a member on websites.	3.63	0.61	Very high
5. Not requesting copies of newly obtained software from friends	3.92	0.31	Very high
6. Not using a friend’s email password to access or read their private messages.	3.98	0.16	Very high
7. Not allowing others to copy or download programs or music obtained from the Internet.	3.94	0.27	Very high
8. Refraining from using offensive language in websites or chat rooms despite online anonymity.	3.81	0.49	Very high
9. Not posting or forwarding messages that may cause harm to others.	3.96	0.22	Very high
Overall	3.89	0.21	Very high

**Table A2:** Psychological problems among participants.

Question	Mean Score	SD	Level
1. Feeling confident when expressing opinions or interacting with others.	1.98	1.19	Low
2. Feeling dissatisfied with oneself and current life circumstances.	2.30	0.99	Low
3. Frequently thinking about the pain resulting from past life mistakes.	2.91	1.12	Low
4. Frequently feeling lonely and isolated.	2.60	1.09	Low
5. Feeling bored with life, including work, study, or social surroundings.	2.60	1.07	Low
6. Frequently feeling irritable and unable to control emotions when situations do not meet expectations.	2.24	1.04	Low
7. Preferring to seek novel and exciting experiences.	3.43	1.03	High
8. Frequently feeling fearful or anxious that situations will not meet expectations.	3.54	1.05	High
Overall	2.70	0.58	Moderate

**Table A3:** Social relationship problems among participants.

Question	Mean Score	SD	Level
1. Feeling that adjusting to social environments is difficult.	2.45	0.95	Low
2. Having a tendency to avoid relationships with others.	2.42	0.97	Low
3. Feeling that interacting or meeting with others is uncomfortable or burdensome.	2.59	1.04	Low
4. Desiring a warmer family relationship than currently experienced.	2.71	1.21	Moderate
5. Frequently thinking about the pain resulting from past life mistakes.	1.84	0.93	Low
6. Feeling that one’s current social relationships are insufficient.	2.10	1.04	Low
Overall	2.35	0.68	Low

**Table A4:** Family relationship levels among participants.

Question	Mean Score	SD	Level
1. Feeling a sense of warmth within the family.	3.60	0.63	Very high
2. Family members consistently show care and attention.	3.64	0.57	Very high
3. When facing serious problems, parents and family members provide support to help resolve them.	3.44	0.71	Very high
4. Parents and family members often praise you when you do something good	3.57	0.63	Very high
5. When you make a mistake, you are often criticized by parents and family members.	2.70	0.94	High
6. Parents and family members express love in ways that you can clearly feel and perceive.	3.66	0.58	Very high
7. You frequently feel satisfied with your parents and family members.	3.24	0.74	High
8. You frequently engage in activities together with your parents and family members.	2.73	0.94	High
9. You would like your relationship with your family members to improve.	2.94	0.96	High
Overall	3.28	0.40	Very high

**Table A5:** Peer relationship levels among participants.

Question	Mean Score	SD	Level
1. Having several close friends.	3.00	0.93	High
2. When going out, you usually spend time with friends rather than others.	3.10	0.90	High
3. When facing problems, you usually seek advice from your friends.	3.08	0.82	High
4. Your friends provide encouragement when you feel discouraged.	3.49	0.64	Very high
5. You feel happy when spending time with friends.	3.60	0.53	Very high
6. You feel that your friends understand you better than anyone else.	2.78	0.70	High
7. You tend to choose friends who demonstrate good conduct.	3.48	0.60	Very high
8. You spend more time with friends than with anyone else.	2.63	0.89	High
9. You often advise your friends when they are about to engage in inappropriate behavior.	3.25	0.63	High
10. You feel deeply upset when you have conflicts or arguments with your friends.	3.45	0.66	Very high
Overall	3.19	0.45	High
